# The Diagnosis and Treatment of Penetrating Wounds of the Abdomen

**Published:** 1908-03

**Authors:** Floyd W. McRae

**Affiliations:** Atlanta, Ga.


### Journal-Record of Medicine

Successor to Atlanta Medical and Surgical Journal, Established 1855, and Southern Medical Record, Established 1870.

**OWNED BY THE ATLANTA MEDICAL JOURNAL CO.**

**Published Monthly.**

*Official Organ Fulton County Medical Society, State Examining Board, Presbyterian Hospital, Etc.*

EDGAR G. BALLENGER, M. D., Editor.
BERNARD WOLFF, M. D., Supervising Editor.
A. W. STIRLING, M. D., C. M., D. P. H., JNO. S. HURT, M. D., Associate Editors.
GEORGE BROWN, M. D., Business Manager.

COLABORATORS

F. W. McRAE, M. D., Surgery.
H. F. HARRIS, M. D., Pathology and Bacteriology.
E. B. BLOCK, M. D., Diseases of the Nervous System.
MICHAEL HOKE, M. D., Orthopedic Surgery.
CYRUS W. STRICKLER, M. D., Legal Medicine and Medical Legislation.
E. C. DAVIS, A. B., M. D., Obstetrics.
E. G. JONES, A. B., M. D., Gynecology.
R. T. DORSEY, Jr., B. S., M. D., Medicine.
J. N. LeCONTE, M. D., Diseases of the Stomach and Intestines.
L. B. CLARKE, M. D., Pediatrics.
EDGAR PAULIN, M. D., Opsonic Medicine.
R. R. DALY, M. D., Medical Society.
A. W. STIRLING, M. D., Etc., Diseases of the Eye, Ear, Nose and Throat.
BERNARD WOLFF, M. D., Diseases of the Skin.
E. G. BALLENGER, M. D., Diseases of the Genito Urinary Organs.

VOL. IX. MARCH, 1908. NO. 12

### ORIGINAL COMMUNICATIONS.

### THE DIAGNOSIS AND TREATMENT OF PENETRATING WOUNDS OF THE ABDOMEN.

(Abstract)

BY FLOYD W. MCRAE, ATLANTA, GA.

Diagnosis.—The diagnosis of penetration of the abdomen, in civil practice, can not be made with even approximate accuracy without tracing the wound. Attempts to folow wounds with the finger or the probe frequently eventuate in failure—neither confirming nor disproving penetration. These are dangerous expedients—universally condemned by the best authorities.

According to the most reliable statistics recorded, in only ten percent. of the cases of penetrating abdominal wounds do the hollow and solid viscera escape serious injury. There is but one positive sign of perforation—seen exceptionally—the escape of gas or feces externally. Shock is a very variable symptom, marked in nervous individuals, often practically absent in men of "iron nerve" in spite of grave lesions. When pronounced and persistent, it is strong presumptive evidence of free bleeding. Nausea, vomiting and muscular tension are signs of value.

Treatment.—In civil life, with fair hospital facilities, aseptic technic, and a competent surgeon available, almost every penetrating wound of the abdomen that does not prove immediately fatal on account of shock and hemorrhage should be dealt with by means of prompt operation. When everything is ready, the preparation of the patient should be made on the operating table during the administration of the anaesthetic; the stomach should be washed out and the field of operation thoroughly prepared. If there is any doubt as to the penetration of the wound, it should be traced. By tracing, is meant laying open the wound of entrance, the wound of exit and the intervening track of the bullet. Any one, or all three may be necessary to prove or disprove penetration of the peritoneal cavity beyond equivocation.

If the wound is penetrating, the operation should be so extended as to constitute an exploratory celiotomy, and if visceral damage is found, immediate repair should be made. Unless very inconveniently located, the wound of entrance should be enlarged sufficiently for thorough exploration.

On opening the abdomen the escape of gas or feces should be noted and immediate search be made for the bleeding vessel or vessels, if there is profuse hemorrhage. Free bleeding should be arrested at once, and if essential to its accomplishment, the incision should be enlarged and the patient partially or completely eviscerated. These extreme measures should be avoided, if possible. Long incisions, evisceration, undue exposure of intestines and rough handling, greatly increase the operative mortality.

The first portion of the intestine encountered should be brought out of the wound and a tape or a strip of gauze thrown round it by pushing one end through the mesentery, between vessels, with a hemostat. The ends of the tape or gauze are caught and held by forceps; and a cross nick is made either above or below to give a definite point of departure. This is a little procedure of great practical value, requiring only a few seconds of time in its accomplishment. To look for the ileocecal junction or some other readily recognizable portion of the intestinal tract is a waste of time and the additional handling tends to spread infection in the presence of perforation with leakage. The whole intestinal tract should be rapidly examined, first above and next below the gauze. Slits in the mesentery should be repaired, bleeding be arrested and intestinal perforations be closed as found.

The perforations should be closed on its convex surface when it can be done without too much contracture of the lumen of the gut, and in its longitudinal axis. Perforations near the mesenteric border usually can be closed best transversely. Less obstruction is offered to the fecal flow by the longitudinal seam than by a transverse one, and the dangers from leakage and obstruction are consequently diminished. Care should be taken to see that the mesenteric vessels supplying the intestine are not interfered with. When the margins of the perforation are ragged and contused, they should be trimmed off. When the wound is small and clean cut, interrupted Lembert sutures are all that is necessary. When the perforation is long and ragged, a few interrupted Czerny sutures should be inserted; over these Lembert sutures of celluloid linen (Pagenstecher) or silk should be placed to close the perforation accurately—care being taken not to infold too much tissue. The intestine in gunshot perforation is often in a state of spasmodic contraction. I have found this condition present in some portion of the alimentary canal in almost every case of gunshot wound that has come under my observation. This contraction, reducing the gut much below its normal caliber, enhances the danger of infolding too much tissue, the bite of the suture being six to eight times deeper than it would have been had the gut retained its normal degree of distention and relaxation.

When there is considerable separation of the gut from its mesentery, resection and end-to-end anastomosis should be done. It is frequently a question of judgment as to whether a large number of perforations, close together, should be sutured separately, or a resection of that portion of the intestine be resorted to. The latter is generally considered to be the better procedure, taking less time and diminishing the danger of obstruction.

Under ordinary conditions, only a small portion of the intestine need be withdrawn from the abdomen at a time; as the surgeon draws out and traces the intestine, his assistant follows after and replaces it in the abdominal cavity. After examining from the point of departure upwards to the end of the gut or vice versa, the opposite portion of the intestine is drawn out by the strip of gauze around it and followed in the same way as above described, dealing with each injury seriatim. The strip of gauze around the intestine is next removed and the wound in the mesentery closed.

Careful handling of the intestine, without undue exposure, is essential in order to secure good results. Evisceration, rough handling and the long exposure of intestine have turned the scales in many of these abdominal injuries and led to a fatal result.

Note should always be taken of the presence or the absence of urine in the peritoneal cavity, and the bladder should be examined for injury. If the bladder has been perforated, it should be sutured accurately by interrupted sutures of catgut, which embrace all the coats down to the mucous membrane. If the suture line can not be tested by distending the bladder, a catheter should be introduced or drainage be obtained through a perineal incision. I prefer continuous catheterization through the urethra to making a perineal section. In these days of clean surgery and skilful nursing one need have little apprehension that the catheter, when properly handled, will cause chills or produce urethral irritation.

In one of my cases, the bladder was tunneled for two inches, but other than the time required in making the repair, it did not complicate matters in the least. Two of the fatal cases in the Grady Hospital statistics, quoted below, were due to failure to find and properly deal with bladder injuries.

Injuries to the solid viscera, other than the kidney and pancreas, are dangerous chiefly on account of the hemorrhage.

Bleeding from the liver can usually be controlled by light gauze packing. Where this does not suffice, deep through-and-through, heavy catgut sutures, loosely tied, or the tape suture of Tiffany, may be necessary.

Superficial injuries to the cortical substance of the kidney should be drained through the loin. It is dangerous to attempt to drain a kidney transperitoneally.

When there is extensive laceration of the kidney substance, and when the renal vessels are severed, a nephrectomy should be done. Temporizing with a kidney thus injured will usually lead to a fatal result

Of first importance in the after treatment of penetrating wounds of the abdomen is rest, general and alimentary. When the injury is in the upper portion of the intestinal tract, nothing should be allowed by the mouth, except sterile water for several days. The lower bowel should be kept unloaded by simple enemata and rectal feeding should be employed. Purgatives should be avoided. A little morphin or codein may be necessary to relieve great pain and to secure proper rest. Opiates, however, should be given very guardedly. Strychnine is usually indicated. When there has been considerable hemorrhage, if practicable, a quantity of normal salt solution should be left in the abdomen.

In the after treatment of these cases, when there is marked gaseous distention, with a tendency to intestinal paresis, alum enemata, as first suggested by the late Virgil O. Harden, of Atlanta, are of great value. Sulphate of salicylate of oserin in doses of one one-hundredth of a grain, every three to six hours, has seemed to have a salutary effect. I absolutely exclude milk and egg diet by the mouth for the first five to eight days. Milk and eggs tend to increase the formation of gas and to constipate the bowels.

Purgatives should be employed with great care and only after four to six days following the operation.

Prognosis.—It is unfair to quote military statistics as an argument against operations in civil life. The conditions under which the injuries are received and the character of the injuries are so different as hardly to admit of just comparison. In modern military practice, the abdominal injuries are due to small calibre, steel jacketed bullets of high velocity, which go directly through, making small, clean cut perforations in the hollow as well as the solid viscera, from which there is very little leakage or hemorrhage. The injuries are more frequently than otherwise received in the standing posture, with comparatively empty intestinal tract, by individuals in the prime of life—picked specimens of physical manhood. In civil practice, the individuals are more frequently than otherwise shot in drunken brawls when the alimentary canal is filled with alcohol and with all sorts of irritating and even infectious material. The wounds are made by infected bullets, which have been carried around in the hip pocket by "pistol toters," of a large calibre and low velocity, that make large, ragged perforations in the hollow viscera and jagged wounds in the mesentery and solid viscera that leak freely and bleed furiously. Other things being equal, the surgeon who opens the abdomen the earliest after receipt of the injury, does his work rapidly and accurately, and who closes the abdomen in the shortest time, will get the best results as to mortality.

The prognosis of penetrating gunshot wounds made by large calibre, low velocity bullets, without operation, is bad. As shown by the statistics of the Crimean War, the American Civil War and civil hospitals, about ninety percent. prove fatal. Stab wounds and wounds made by small shot, or small calibre, steel jacketed, high velocity bullets are less fatal, giving a mortality of from fifty to seventy-five percent. Wounds of the umbilical and hypogastric regions are peculiarly dangerous. In these regions the small intestines rarely escape perforation.

| PENETRATING ABDOMINAL WOUNDS. | Number | Deaths | Mortality Per Cent. |
|---|---|---|---|
| Crimean War | 241 | 222 | 92.1 |
| Italian War | 246 | 163 | 87.2 |
| Civil War | 3717 | 3331 | 87.2 |
| Franco-Prussian War | 1047 | 784 | 74.8 |
| Spanish-American War | 116 | 81 | 69.8 |

Of the 113 cases in the Spanish-American war, 13 cases had operations with 11 deaths—a mortality of 84.6 per cent. Of the 193 cases not operated upon, 79 died—a mortality of 67.9 per cent.

The contents of the stomach are comparatively innoccuous. The virulence of the infection contained in the alimentary canal progressively increases to the ileocecal junction; the contents of the large intestine are less infectious, and wounds of the large intestine are less fatal than those of the small intestine.

| Penetrating Abdominal Wounds | Operation | Deaths | Mortality Per Cent. | No Operation | Deaths | Mortality Per Cent. |
|---|---|---|---|---|---|---|
| Grady Hospital cases | 31 | 8 | 25.4 | 17 | 10 | 25.4 |
| McRae's private cases | 9 | 3 | 33.3 | 5 | 4 | 80. |

In looking over the statistics of the Grady Hospital, I find that there have been since July, 1899, forty-eight cases of penetrating wounds of the abdomen. Thirty-one of these were operated upon, with a mortality of 25.4 per cent. Thirteen were not operated upon, with a mortality of 58 per cent. Not a single death could have been attributed to the operation perital. In one fatal case, a perforation of the gall bladder was overlooked. In two fatal cases, injuries to the urinary bladder were overlooked. In two cases, the deaths were evidently due to kidney injuries, with leakage of urine into the peritoneal cavity, which were overlooked. In one case, intestinal perforations were overlooked. In another case, the abdomen was opened, found filled with blood, the blood washed out and the abdomen closed without finding the source of hemorrhage, the patient subsequently dying from internal hemorrhage.

These thirty-one operations have been done by eight different surgeons and three house surgeons (in turn); this large number of operators giving only a fair average of surgical skill. In spite of the fatalities due to overlooked injuries, the statistics following operations have been very gratifying and have constantly improved. The cases not operated upon were either very bad cases, or those in which the injury was in such a locality as to indicate no injury to important viscera. The average of the cases operated upon has been worse than those treated expectantly.

My personal experience and responsibility cover but fourteen cases. I have, however, had intimate knowledge of most of the forty-eight Grady Hospital cases recorded below and have assisted in, or been present at many of the operations done by other surgeons and have followed up their cases in the wards. I have been forced by these observations and experience from an attitude of passive expectancy to a position of earnest advocacy of early operation in all penetrating wounds of the abdomen, unless positively contraindicated by the individual's physical condition and surroundings, or the absence of a competent abdominal surgeon and assistants.

Of my fourteen cases, seven had the benefit of early operation, with six recoveries. The percentage of recoveries was 85.7. Five of the seven operations were for rifle or pistol shot wounds and all presented grave visceral lesions. One had an extensive laceration of the liver, with excessive hemorrhage, the patient exsanguinated and pulseless at wrist—result, recovery. One had three perforations in stomach with leakage—recovery. Three had multiple perforations in the small intestines with leakage in all—two recoveries, one death. In one of the above, the bladder was also injured—recovery.

Two operations were done for stab wounds of the liver. Free hemorrhage in both cases was controlled by suture of the liver wounds and gauze packing—both recovered.

I have treated four pistol shot wounds of the abdomen expectantly, with three deaths and one recovery.

One man with a stab wound treated expectantly, died. Five cases, four deaths, one recovery—80 mortality. I have done two late operations for complications due to penetrating wounds that might have been prevented by early operation. Both died—mortality 100 percent.

The above statistics, my personal experience, observation of our Grady Hospital cases, and a careful review of the literature on penetrating wounds of the abdomen, abstracted below, have led me to make the following conclusions as to civil practice:

In civil practice, every suspected penetrating wound of the abdomen, under favorable conditions of practice, should have the benefit of wound tracing.

When the wound proves to be penetrating, an exploratory laparotomy should be done at once and visceral damage excluded or repaired as far as practicable.

There is far less danger from wound tracing than there is from probing or from masterly inactivity, while awaiting positive evidence of visceral damage requiring operative interference.

Local toilet with moist sponging for cleansing is better than free peritoneal irrigation.

When in doubt, it is safer to drain.

Operations done within two hours should not give a mortality over 25 to 30 percent., within four hours, over 40, within six hours, over 50, within eight hours, over 60, within twelve hours, over 70 percent.

After twelve hours, expectant treatment is best, unless there is some definite indication for operation.

The above deductions do not apply to military practice. Expectant treatment in military practice has given better results with wounds made by modern arms than has operative treatment.

